# Post‐Traumatic Bronchial Artery Pseudoaneurysm: A Case Report

**DOI:** 10.1155/crra/3331859

**Published:** 2026-08-31

**Authors:** Maliha Majeed, Erin Moffett, Dennis Wulfeck

**Affiliations:** ^1^ Department of Diagnostic Radiology, HCA Healthcare/USF Morsani College of Medicine GME/HCA Florida Trinity Hospital, Trinity, Florida, USA; ^2^ Department of Diagnostic Radiology, MBB Radiology/Radiology Partners, Jacksonville, Florida, USA

**Keywords:** bronchial artery, case report, coil embolization, pseudoaneurysm, thoracic spinal fracture

## Abstract

A 34‐year‐old woman with a history of Turner syndrome presented to the emergency department after a high‐speed motor vehicle collision. Computed tomography (CT) of the thoracic spine demonstrated an unstable inferior endplate fracture of T7 with bilateral laminar fractures. Computed tomography angiography (CTA) of the chest and magnetic resonance angiography (MRA) of the aorta demonstrated a 6‐mm focal outpouching of contrast along the right aspect of the descending thoracic aorta, concerning for an unruptured bronchial artery pseudoartery aneurysm. Digital subtraction angiography (DSA) confirmed a pseudoaneurysm arising from the origin of the right bronchial artery, which was successfully treated with coil embolization.

## 1. Case

We present a rare case of a 34‐year‐old woman with a past medical history significant for Turner syndrome who presented to the emergency department following a high‐speed rear‐end motor vehicle collision while stationary on the highway. She required extrication from the vehicle and was nonambulatory at the scene. On arrival to the emergency department, the patient was mildly confused and was tender to palpation along the thoracic spine but was able to move all extremities.

**Figure 1:**
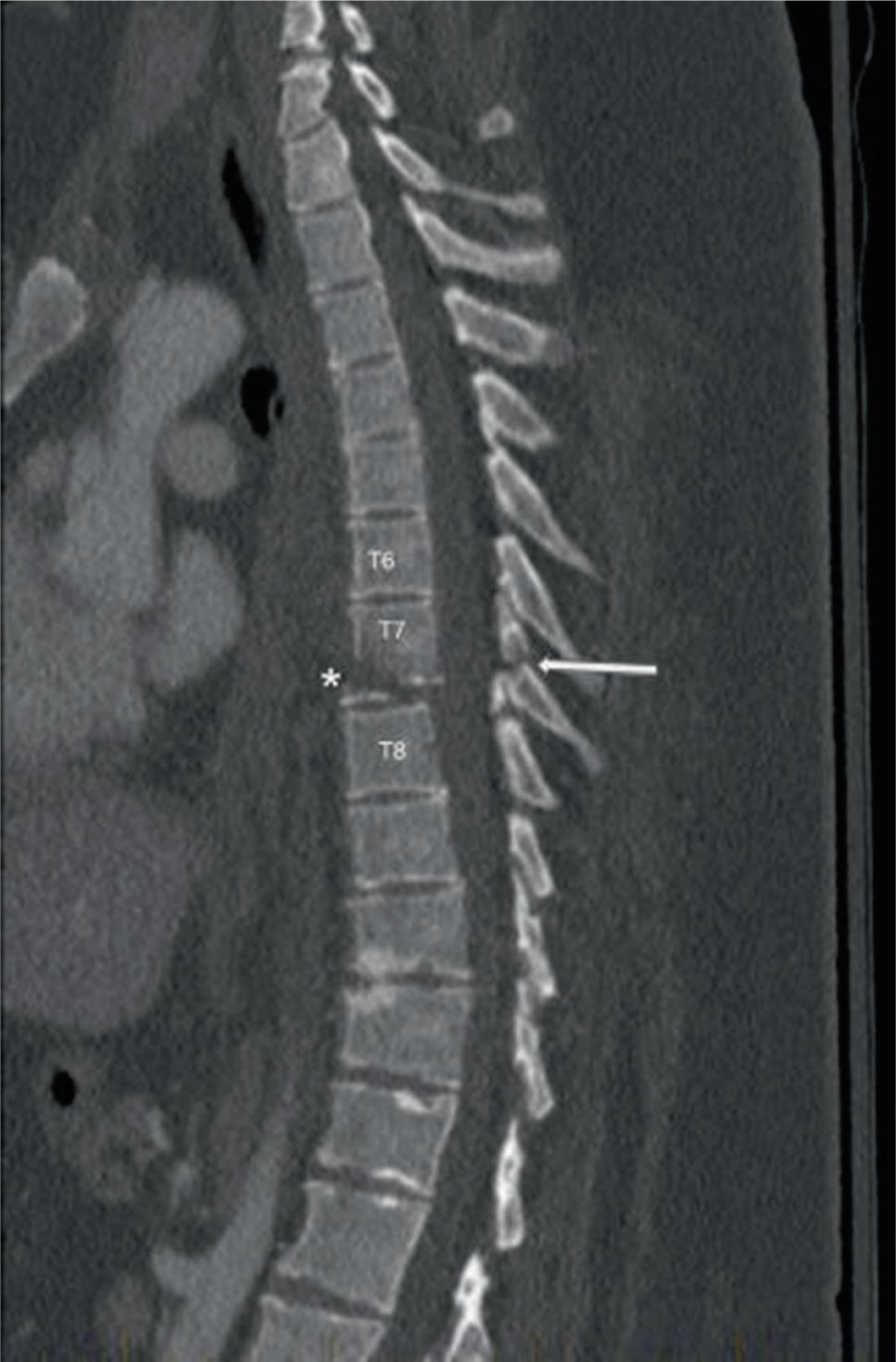
Sagittal thoracic spine computed tomography demonstrates acute displaced fractures involving the inferior endplate ( ^∗^) and the right lamina of T7 (←). Additional fracture of the left T7 lamina is not shown here. Grade I retrolisthesis of T7 on T8 is also noted with up to 7 mm of narrowing of the spinal canal.

**Figure 2:**
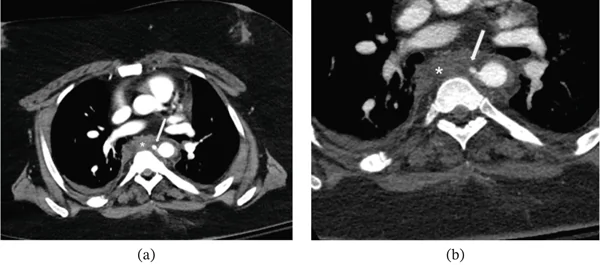
(a and b) Axial computed tomography angiography of the chest demonstrates a prevertebral hematoma at the level of T6 ( ^∗^). Image 2b represents a slightly zoomed‐in view to better visualize the 6‐mm contrast‐filled outpouching arising from the right lateral aspect of the descending thoracic aorta (←).

**Figure 3:**
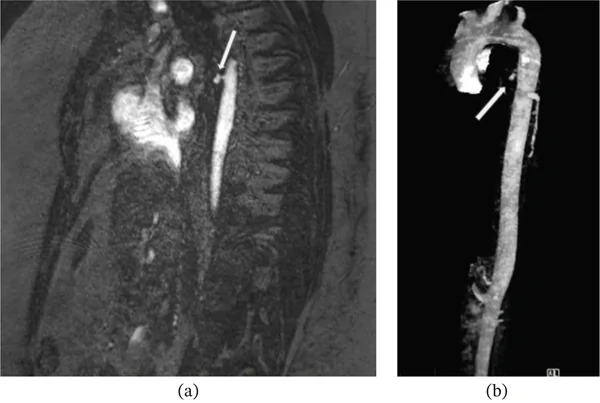
(a) Sagittal postcontrast T1‐weighted imaging magnetic resonance angiography of the thoracic aorta and (b) volume‐rendered 3D reconstruction demonstrate abnormal focal dilation at the origin of the right bronchial artery at the level of T6 (←).

CT of the thoracic spine demonstrated an inferior endplate fracture of T7 with Grade 1 retrolisthesis of T7 on T8, bilateral T7 laminar fractures, and associated spinal canal narrowing up to 7 mm (Figure 1). CTA of the chest demonstrated a well‐circumscribed outpouching of contrast arising along the right side of the descending thoracic aorta with an adjacent small posterior mediastinal hematoma (Figure 2a,b). Subsequent contrast‐enhanced MRA confirmed a 6‐mm pseudoaneurysm at the origin of the right bronchial artery at the level of T6 (Figure 3a,b). The patient remained hemodynamically stable with a hemoglobin of 14 g/dL and without symptoms suggestive of pseudoaneurysm rupture.

**Figure 4:**
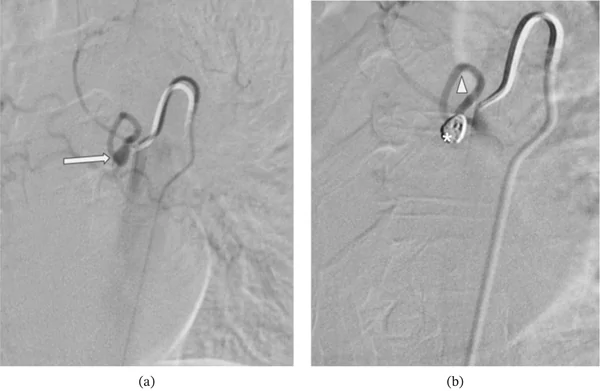
(a) Pretreatment catheter‐based digital subtraction angiography confirms a pseudoaneurysm arising from the bronchial artery just outside the aortic wall (←), and (b) postembolization digital subtraction angiography shows complete embolization of the pseudoaneurysm ( ^∗^) with maintained flow within the bronchial artery (△).

Bronchial angiography demonstrated a pseudoaneurysm arising just external to the aortic wall (Figure 4a). Coil embolization was performed, and the pseudoaneurysm was successfully occluded (Figure 4b). The patient then underwent percutaneous instrumentation with closed reduction for stabilization of the T7 fracture. Her postoperative course was uncomplicated, and she was discharged to a rehabilitation facility.

## 2. Discussion

Thoracic spine fractures, particularly those resulting from high‐energy trauma, are frequently associated with vascular injuries due to the close anatomic relationship between the vertebral column and the mediastinum [1]. While traumatic aortic injury is the most widely recognized vascular association, smaller branch vessel injuries, such as injuries to the intercostal arteries and bronchial arteries, can also occur and may be overlooked in hemodynamically stable patients [2–4].

Bronchial artery pseudoaneurysms are exceedingly rare but potentially life‐threatening vascular abnormalities, with fewer than 60 cases reported in the literature [4]. Unlike true aneurysms, which involve dilation of all three layers of the vessel wall, pseudoaneurysms result from a focal disruption of the vessel wall resulting in thrombus accumulation between the tunica media and tunica adventitia [5, 6].

Bronchial artery pseudoaneurysm may be an incidental finding on contrast‐enhanced CT or CTA, especially in hemodynamically stable or asymptomatic patients [7]. They most commonly occur secondary to trauma, as in our case, although other etiologies including infection, vasculitis, connective tissue disorders, and iatrogenic causes are also important etiologies [8]. Because pseudoaneurysms lack structural wall integrity, they carry a substantial risk of rupture, more so than true aneurysms [9]. Rupture can result in life‐threatening hemorrhage into the lungs and/or mediastinum which may present as hemorrhagic shock, hemoptysis, or compressive symptoms such as dysphagia or superior vena cava syndrome [10].

Early identification and prompt management of bronchial pseudoaneurysms are critical. In the trauma setting, clinical signs are unreliable, and emphasis is placed on diagnostic imaging [11]. CTA is considered the first‐line imaging modality for evaluation of suspected vascular injury due to its rapid acquisition, high spatial resolution, and widespread availability [12]. Findings that should prompt CTA include high‐energy mechanism of injury, thoracic vertebral fractures, mediastinal hematoma, or unexplained hemothorax [11, 12]. CTA with delayed imaging can be obtained to assess for active contrast extravasation in the setting of hemodynamic instability. Digital subtraction angiography (DSA) remains the gold standard of imaging due to the ability to evaluate in real time and therapeutic potential [4]. MRA may be considered in patients with a contraindication to iodinated contrast [12, 13]. However, the long‐acquisition time of MRA and the presence of MR‐incompatible medical equipment may be prohibitive in many trauma patients [13]. MRA was performed in our case to further evaluate a possible Type A Stanford aortic dissection versus flow artifact noted on the initial CTA. This was ultimately deemed artifactual.

Treatment of bronchial artery pseudoaneurysms via transcatheter embolization with coils, polyvinyl alcohol (PVA), gel foam, or a combination is considered highly effective and less invasive than an open surgical approach [14]. However, in cases of embolization failure or rapidly progressive vascular compromise, surgical management may be preferred [14].

This report describes a single case of a rare vascular complication, which limits the generalizability of management decisions. The optimal management approach may depend on patient stability, institutional resources, and operator experience. Additionally, due to the rarity of bronchial artery pseudoaneurysms, there are no standard guidelines for management. This case highlights the importance of maintaining a high index of suspicion for bronchial artery aneurysms or pseudoaneurysms in patients with thoracic spinal trauma.

This case highlights a rare but potentially life‐threatening complication of thoracic spine trauma. Maintaining a high index of suspicion, appropriate use of multimodality imaging, and timely endovascular intervention are critical to successful management.

## Funding

This study was supported by HCA Healthcare, 10.13039/100016646.

## Ethics Statement

This case report was written in accordance with the 2013 CARE (CAse REport) guidelines.

## Consent

Informed consent was not obtained from the patient for publication of this case report. In accordance with the recommendations of the International Committee of Medical Journal Editors (ICMJE), this report does not include any personally identifiable information. All imaging data and clinical details have been fully anonymized such that the patient cannot be identified through the text, images, or any other material presented herein. No identifying details—including names, dates of birth, hospital or medical record numbers, or other protected health information—are disclosed. Given the anonymized and nonidentifiable nature of the material, a formal consent waiver was deemed appropriate in compliance with ICMJE guidelines for the protection of research participants.

## Conflicts of Interest

The authors declare no conflicts of interest.

## Data Availability

Data sharing not applicable to this article as no datasets were generated or analyzed during the current study.
